# The experiences of clinical nurses coping with patient death in the context of rising hospital deaths in China: a qualitative study

**DOI:** 10.1186/s12904-022-01054-8

**Published:** 2022-09-22

**Authors:** Jinxin Zhang, Yingjuan Cao, Mingzhu Su, Joyce Cheng, Nengliang Yao

**Affiliations:** 1 Centre for Health Management and Policy Research, School of Public Health, Cheeloo College of Medicine, Shandong University, Jinan, China; 2 NHC Key Lab of Health Economics and Policy Research, Shandong University, Jinan, China; 3 School of Nursing and Rehabilitation, Cheeloo College of Medicine, Shandong University, Jinan, China; 4 Department of Nursing, Qilu Hospital, Cheeloo College of Medicine, Shandong University, Jinan, China; 5 Johns Hopkins University School of Medicine, Baltimore, United States

**Keywords:** Clinical Nurse, Hospitals, Death, Qualitative

## Abstract

**Background:**

Chinese clinical nurses are increasingly confronting patient death, as the proportion of hospital deaths is growing. Witnessing patient suffering and death is stressful, and failure to cope with this challenge may result in decreased well-being of nurses and impediment of the provision of “good death” care for patients and their families. To our knowledge, few studies have specifically explored clinical nurses’ experiences coping with patient death in mainland China.

**Objective:**

We aimed to explore nurses’ experiences coping with patient death in China in order to support frontline clinical nurses effectively and guide the government in improving hospice care policy.

**Methods:**

Clinical nurses were recruited using purposive and snowball sampling between June 2020 and August 2020. We gathered experiences of clinical nurses who have coped with patient death using face-to-face, semi-structured, in-depth interviews. Audio recordings were transcribed verbatim and analyzed using thematic analysis.

**Results:**

Three thematic categories were generated from data analysis. The first was “negative emotions from contextual challenges.” This category involved grief over deaths of younger persons, pity for deaths without family, and dread related to coping with patient death on night duty. The second category was “awareness of mortality on its own.” Subthemes included the ideas that death means that everything stops being and good living is important because we all die and disappear. The third category was “coping style.” This category included focusing on treating dying patients, recording the signs and symptoms, and responding to changes in the patient’s condition. It also involved subthemes such as avoiding talk about death due to the grief associated with dying and death, and seeking help from colleagues.

**Conclusions:**

Clinical nurses’ emotional experiences are shaped by intense Chinese filial love, charity, and cultural attitudes towards death. Reasonable nurse scheduling to ensure patient and staff safety is a major priority. “Good death” decisions based on Chinese ethical and moral beliefs must be embedded throughout hospital care.

**Supplementary Information:**

The online version contains supplementary material available at 10.1186/s12904-022-01054-8.

## Background

Patient death is an inevitable occurrence in many medical facilities, where nurses are vital in coordinating and providing hospital care. Chinese clinical nurses have had to increasingly confront patient death, as the proportion of patients dying in hospitals is growing. As a whole, hospital deaths rose from 20.20% in 2009 to 22.5% in 2017 [1]. To our knowledge, health resources in China are skewed toward hospitals rather than the primary health care sector [2]. The number of hospital beds grew dramatically from 2.3 per 1,000 people in 2003 to 5.7 per 1,000 people in 2017, and the hospitalization rate increased substantially (3.6% to 17.6%) from 2003 to 2017 [2, 3]. Due to the unprecedented accessibility and availability of hospital beds, the number of patients dying in hospitals has grown accordingly in recent years [4–6]. In addition, the Chinese government has been promoting hospice care delivery for the past few years, motivating many departments in general hospitals to set up “hospice care wards.” Hospitalization rates for patients requiring hospice care have correspondingly increased, thus increasing the likelihood of hospital death [7, 8]. Moreover, home medical resources in China are still largely inadequate and inaccessible, but the proportion of homebound patients in China is increasing [9, 10]. Unbearable pain often forces homebound patients and their families to seek help from medical facilities [11], which increases the possibility of hospital death [8]. Therefore, clinical nurses must be prepared to face patient death and cope appropriately, to facilitate a “good death” for the patients and family members [12]. The concept of a “good death” is based on the principles of alleviating pain and suffering and preserving dignity, through practices such as preparing for death and saying farewell [13]. The “good death” is a critical concept in hospice and palliative care [14].

Clinical nurses are vital resources for every country. Their health and safety are crucial for healing illnesses, saving patient lives, and delivering “good death” care [5]. However, clinical nurses have been found to face overwhelming psychological stress and negative attitudes toward death due to their experiences with patient death, and the majority of them felt unprepared [15–17]. When health-care providers fail to cope with challenges related to patient death, they experience burnout, low job satisfaction, poor work performance, high staff turnover, decreased quality of care, and severely compromised well-being [12, 18, 19]. Some observational studies have revealed the importance of healthcare professionals’ perspectives on meaning in life, self-care, self-awareness, communication with patients and families, clinical experience, and palliative care training [20–22]. Other recent interventional research has shown that workshops and end-of-life care models can enhance professionals’ competence in coping with death-related issues [23]. However, those studies did not target the experience of mainland China’s frontline clinical nurses’ experiences coping with patient death.

In China, discussing death remains taboo, due to the influence of traditional philosophical concepts. Several studies have shown that care providers appear to lack confidence in the communication of cultural preferences about death-related issues [24]. Traditional perspectives view death as unfortunate or mysterious. Thus, open conversation about dying and death is rare [19, 25, 26]. When patients and their families face imminent death, they may become especially emotionally desperate, and avoid discussing death or end-of-life care plans [27, 28]. Given this hesitancy toward discussing death openly, nurses rarely have the opportunity to provide honest and timely disclosure of death-related information. This makes it more difficult for patients and their relatives to achieve a “good death.”

The National Health Commission of China strongly encourages the provision of high-quality end-of-life care services for older adults, as a method of upholding filial piety [29]. China is also fundamentally a Humanist society, and most Chinese people prefer to believe in their own capacity to control their lives rather than the influence of supernatural beings, which shapes their views on life and death. To date, studies on the experiences of clinical nurses coping with patient death are still limited. Such evidence-based knowledge is crucial for supporting frontline clinical nurses effectively and guiding the government to enact hospice care responses to achieve a “good death” for the patient. Therefore, we aimed to describe the experiences of coping with patient death among frontline clinical nurses in mainland China.

## Methods

### Study design and participants

We conducted qualitative research through face-to-face interviews. We captured details about clinical nurses’ experiences coping with patient death in two large-scale, tertiary, comprehensive public hospitals (Hospital 1 and 2), which integrate medical treatment, scientific research, teaching, prevention, and health care [30]. Hospital 1 has more than 5,100 hospital beds, and Hospital 2 has 4.4 million outpatient and emergency visits annually. Both hospitals have undergone significant expansions in recent years.

Clinical nurses who met the criteria were selected through purposive sampling and snowball sampling. Inclusion criteria included: (1) fully qualified clinical nurses currently working in her/his unit; (2) engaged in frontline clinical nursing work; (3) relevant experience in dealing with patients who died within the past six months; (4) willingness to cooperate in completing the interview. Exclusion criteria included: (1) those not providing direct care to the patient, such as management and/or clerical workers, and (2) nursing students in practice. The interview outline was revised based on a literature review and team discussion. Data saturation [31], or the point at which no new themes were generated from respondents’ experiences, determined the sample size. The interviewers knew the first interviewee through a common mentor. The rest were recruited through snowball sampling. We also used purposive sampling for controlling heterogeneity in variation in years of work experience, previous experiences coping with patient death, and department of employment.

### Procedures

Our research procedures were performed in accordance with the regulations and principles of the Ethics Review Board at the Shandong University’s School of Nursing and Rehabilitation (e.g., Privacy and Confidentiality; Informed Consent). Before the interview, we explained the objectives and voluntary nature of the study to the participants and obtained a signed consent form from each participant.

Semi-structured, in-depth, face-to-face interviews were conducted in Mandarin Chinese between June 2020 and August 2020. With the consent of the participants, we audio-recorded and took notes during the interview. The lead author conducted the interviews, which included obtaining participants’ demographic data (age, gender, educational status, work department, years of work experience, and experience in hospice care training at the start of the interview). The interviewer also asked one broad question: “Please tell me about your experiences of coping with patients’ death” and followed up with additional open-ended questions for detailed descriptions: “What thoughts do you generate when you face patient death,” “How do you feel about the patient’s death and their families,” “What challenges do you encounter,” “What did coping with the patient’s death mean to you,” and “What knowledge and competencies do you need to cope with patient death?” The interviewer asked clarification questions when necessary, such as, “Could you please give me an example” and probing questions such as, “Could you please describe more details about that?” Another graduate student from the research team took notes by writing down keywords or short sentences when interviewing. The notes were used as a reference during data analysis.

During data collection, we begin to note the potential significance of certain data and issues of interest, to guide our following analysis. All recordings were transcribed verbatim by two interviewers within 24 hours of the interview. The unpaid interview was conducted during the participant’s work (paid) time. Two interviewers were trained in qualitative research methods and had prior interview experience.

### Data analysis

Themes were mainly generated by two researchers; the first author used to be a clinical nurse and was working for her master’s degree in China at the time. The inductive thematic analysis was used for data analysis [32]. Two researchers analyzed the data, which involved repeated readings of the transcribed text to gain familiarity and understanding of all aspects conveyed; generating initial codes related to nurses’ experiences coping with patient death by manually coding using colored pens; reviewing codes that needed to be merged, refined, separated, or discarded; gathering codes with common characteristics to form categories and organizing categories into a meaningful theme; and checking that the themes were relevant to the code and completing sorting of the data. Continuous analysis was used to refine the details of each theme and to restate them in general terms or phrases.

Since researchers are instruments for data generation and analysis, it is essential to consider how subjectivity affects research results. Themes were derived from the data. To ensure the validity and reliability of our results, two researchers separately transcribed and analyzed the data, strictly following the thematic analysis method [32, 33]. Initial themes were communicated with clinical nurses in one-on-one telephone meetings between November 12 and November 19, 2020, and nurses could request changes by clarifying what had been expressed or providing more information. During the data analysis process, researchers thoroughly discussed the coding decisions, categories, and themes until they reached a consensus. All quotes were translated by the lead author into English and back-translated by another graduate student to ensure that they preserved the original intended meaning.

Confidentiality was assured by developing an assigned code number list rather than using participants’ names (e.g., nurse N1, N2, etc.). The anonymity of participants was ensured by removing identifying information from the transcripts. All recordings and texts were kept on a password-protected computer. We reported results following the consolidated criteria for reporting qualitative research (COREQ) checklist [34].

## Results

Our sample consisted of fifteen clinical nurses recruited from two large-scale, tertiary comprehensive public hospitals in Shandong province between June and August of 2020. All participants had experience in coping with patient death in the hospitals at which they were employed within the prior six months. Only 1 of the 15 clinical nurses had participated in hospice care training, and none of the nurses had any experience in death education before the interview. Their time working on clinical nurses’ respective wards before the interviews ranged from 2 to 20 years. The duration of the interviews ranged from 30 to 90 minutes (mean duration: 60 min). Table 1 summarizes the characteristics of the participants.

**Table 1:** Characteristics of participants

**No**	**Department**	**Gender**	**Age, years**	**Work experience, years**	**Final graduation**
**N1**	emergency ward	Female	29	4	Bachelor degree
**N2**	emergency ward	Male	26	2	Bachelor degree
**N3**	emergency ward	Female	38	16	Bachelor degree
**N4**	emergency ward	Male	27	4	Bachelor degree
**N5**	emergency ward	Male	26	4	Bachelor degree
**N6**	oncology department	Female	31	5	Bachelor degree
**N7**	oncology department	Female	40	15	Bachelor degree
**N8**	oncology department	Female	30	7	Bachelor degree
**N9**	oncology department	Female	28	4	Bachelor degree
**N10**	oncology department	Female	30	7	Master degree
**N11**	oncology department	Female	34	11	Bachelor degree
**N12**	ICU	Female	40	20	Bachelor degree
**N13**	ICU	Female	42	15	Bachelor degree
**N14**	ICU	Female	27	2	Master degree
**N15**	ICU	Female	28	5	Bachelor degree

Three thematic categories emerged from the rich interview data provided by 15 clinical nurses (Table 2): negative emotions from contextual challenges; the awareness of mortality on its own; and coping style.

**Table 2:** Theme categories and clusters

**Theme**	**Sub-theme**
**1. Negative emotions from contextual challenges**	A. Grief over deaths of younger persons
	B. Pity for deaths without family
	C. Dread related to coping with patient death on night duty
**2. The awareness of mortality on its own**	A. Death means that everything stops being
	B. Good living
**3. Coping style**	A. Focusing on treating dying patients, recording the signs and symptoms, and responding to changes in the patient’s condition
	B. Avoiding talking about death due to the grief associated with dying and death
	C. Seeking help from colleagues

The first subtheme within the first thematic category identified was “grief over deaths of younger persons”. Thirteen of the 15 participants felt despair when coping with deaths of infants, children, and young and middle-aged patients. The end of a younger person’s life felt more distressing and stressful for clinical nurses than the death of an older person. They associated the deceased with their own children or loved ones, and felt responsible for their deaths.

N1: “A little boy patient in our department passed away. So, all the people in the whole department were in a very low mood for an afternoon.”

N6: “I see the dying boy, and I think of my son... He [dying boy] makes me feel very sorrowful.”

N8: “We feel that the death of the elderly is a natural part of our life. But the [accidental] death of young people made me feel very sympathetic; it’s difficult for me to witness.”

We named the second subtheme “pity for deaths without family”. Nurses’ sadness and regret often emerged from the surrounding context of dying patients and the state of their death. Nurses felt that dying people need a caring environment, and the company of families could allow the patient to die peacefully and achieve a good death. Dying unaccompanied was associated with feelings of sorrow and regret.

N9: “Some patients' family members prepared shrouds for the patient in advance and accompanied the patient until the patient passed away... But when another patient died, his son was abroad and couldn't come back to see him one last time. We sincerely regret it.”

N10: “I was sorting through the patient's belongings when I saw the picture of his kid in his wallet, and I felt so sorry. He [the patient] probably misses his son a lot.”

N15: “How heartbreaking it is that the man had only a care worker with him at the end of his life.”

The third subtheme identified was “dread related to coping with patient death on night duty”. Participants were afraid to cope with death on night shifts. Their fear was related to the heavier workload due to increased hospitalizations and emergency department visits. In large general hospitals, the number of night nurses is much smaller than the number of day nurses. At night, in addition to routine treatment for hospitalized patients, nurses also had to handle new patients’ treatments. Insufficient nursing staff made coping with patient death during the night shift much more difficult.

N2: “Everyone’s energy is limited, but every patient needs attention and needs to be taken care of. I am very scared that I would have to cope with patient death at night. I’m feeling overwhelmed and worried.”

N3: “The administrative department has stipulated that inpatients within the scope of the nurses’ departments cannot be rejected, so the number of patients who come to the hospital to die has also increased in recent years. Clinical nurses on rotating night shifts face tremendous work pressure.”

N14: “Our department did not handle this kind of case [planned deaths in hospital per patient/family request] before, but now we are not allowed to refuse to admit these dying patients. The number of personnel on duty varies between day shifts and night shifts. Due to the surge of [dying] patients, I can be exhausted sometimes when I work nights.”

The second thematic category, “the awareness of mortality on its own”, consisted of two clusters relating to “death means that everything stops being” and “good living”. None of the nurses had previous experience with education regarding death of patients. Their understanding of death was simply that the patient no longer existed and death is nothingness. On the other hand, most clinical nurses recognized that death is an unavoidable part of life and grasped the finality of death.

N7: “After he died, nothing he ever owned mattered. It doesn’t have much to do with his life.”

N12: “After he [patient] dies, there’s nothing left. Deaths from this disease seem inevitable. We had long expected this outcome [death] of his illness.”

Most of the clinical nurses, who were mothers with children, believed that the lessons learned from coping with patient death were living peacefully and living well. Most of them suggesting that they seem to prefer to believe in their own ability rather than the ability of supernatural beings to protect and save them, processing patient death may influence nurses to prioritize good living in daily life.

N6: “We seem to prefer to believe in our own ability rather than the ability of supernatural beings to protect us... It [coping with death] reminds me to live well. I have my children, I can’t get sick, I can’t die. I can’t take care of my son if something happens to me.”

N8: “Death is an unavoidable part of life. I focus on the present and learn to live here and now, especially for my children.”

The third thematic category, “coping style”, consisted of three subthemes: “focusing on treating dying patients, recording the signs and symptoms, and responding to changes in the patient’s condition;” “avoiding talking about death due to the grief associated with dying and death;” and “seeking help from colleagues”. The overwhelming majority of nurses believed that providing direct care was the best way to meet the preferences of patients and their families in the hospital, unless their family instructed them to give up on curative treatment. Participants reported closely observing patients’ vital signs and symptoms, monitoring changes in their conditions, and taking proper care of patients (e.g., providing emergency nursing and palliative care). Therefore, clinical nurses offered multiple life support techniques, including medications, injections, and other treatments intended to cure. Participants felt that their patients’ conditions could result in severe consequences, such as rapid deterioration or death, if not addressed promptly.

N2: “In the clinical setting, we pay attention to assessing the dying patients’ physical conditions, and appropriate actions should be taken immediately if any abnormalities in vital signs appear.”

N4: “They [patients and their families] seem to prefer that we provide her [patient] with medication and injections because that means there is still hope for a cure. I had proficient clinical skills, and I could take good care of patients.”

N5: “Clinical nurses had crucial roles in treating dying patients and keeping them safe. Every month, we need to be trained in first aid and other nursing skills. We [doctor and the nurse] coordinated well in saving the patient.”

N11: “We emphasized the need for their [patients and their families] demand for treatment and integrated aggressive treatment into their end-of-life care.”

Clinical nurses were often unwilling to talk about death with others. Moreover, most participants noted that the underlying cause of being hesitant to talk about death was the grief related to dying and death, rather than feeling like death is mysterious. Only one clinical nurse responded that death brings bad luck, a belief that seemed to have been deeply affected by her superstitious grandmother. Nurses actively resist being engulfed by grief and sorrow, because death means that everything no longer exists.

N7: “I don’t like to talk about death, because I don’t want to immerse myself in heavy topics.”

N9: “When a patient dies, we don't go back into the patient's room that day... I fear that I’d become sad if we talked in depth about dying and death.”

N13: “I was reluctant to engross myself in talking about death with my kid. Little or no attention seems to have been given to talking in-depth about death because of the grave sadness.”

Clinical nurses often sought help from colleagues in situations such as resuscitating actively dying patients, delivering bad news to patients, showing more care in treating dying patients, or deciding when it was and was not appropriate for them to communicate directly with patients’ family members.

N10: “When we encounter difficulties in communicating with dying patients, we usually consult senior nurses... If I take the initiative to comfort the patient, it’s like deliberately reminding the patient that you’re going to die, you’re going to die. But if I don’t comfort the patient, we seem too indifferent. So, I don’t know how to communicate with patients or their families.”

N13: “Whether or not a patient is an organ donor is also essential, and I'd like to be able to participate in talks. I usually ask the attending physician for help because many things need to be conveyed by the doctor, and there will be better results [compared to information being conveyed by nurses].”

## Discussion

We conducted 15 face-to-face individual interviews with clinical nurses (12 female, 3 male). Clinical nurses described a variety of details that shaped their experiences of coping with patients’ death. We identified three main themes, including the negative emotions from contextual challenges, the awareness of mortality on its own, and coping style. Unlike palliative care professionals or nursing students who have been reported to experience fear, burnout, compassion fatigue, and other negative outcomes during coping with patient death [15, 21], clinical nurses’ negative experiences were more focused on the contextual challenges, mainly related to patients’ situations and surroundings, such as excruciating unnatural deaths, unaccompanied deaths of the elderly, and deaths of children and young adults. In China, there are traditional sayings such as “honor old people as we do our own aged parents, and care for other’s children as one’s own,” and “live and let live”. Encountered with dying children, young patients, or unaccompanied elderly patients, clinical nurses showed consideration for the feelings of others by experiencing sadness and regret. These emotional experiences were shaped by strong feelings of extended filial love and charity. As shown in previous studies, the ultimate moral purpose of filial piety in Chinese culture is to extend love for parents and loved ones to the society and even the whole of humanity [35, 36]. This may partly support why nurses have negative emotions when coping with those who die young or unaccompanied. Chinese cultural traditions contain rich ethical and moral principles (e.g., humaneness, humanity, and benevolence) [35, 36]. These values can be used to formulate a vision of a good death contextual model in hospitals that upholds the cultural sensitivities of nurses in China.

Clinical nurses reported that they were afraid to cope with patient death during the night shift, and that they felt stressful and exhausted. As the number of beds in Chinese hospitals continues to increase dramatically and the number of late-life hospitalizations continues to grow [7], many problems have been largely overlooked, such as the shortage of health care workers, unbalanced skill levels, and inadequate work ability of clinical nurses [37, 38]. According to previous studies [39, 40], each clinical nurse has to care for an average of 23 patients at night. In terms of different departments, the nurse to patient (NTP) ratio, or the average number of patients assigned to a nurse, is about 1:3 in ICUs, 1:34 in the oncology department, and about 1:24 in geriatrics overall. China's nurse density of 1.9 per 1,000 is only one-fifth that of Britain and the US [40]. A wide body of international evidence has reported an association between lower nurse staffing levels and higher hospital mortality rates and other adverse patient outcomes [41–44]. Reasonable nurse scheduling to ensure patient and staff safety is a major priority. To our knowledge, the night shift schedule in Chinese public hospitals is comprised of one or more nurses per shift, working 8-12 hours each. In addition, departments with many critically ill patients (e.g., cardiovascular medicine, neurology, neurosurgery, etc.) with already heavy nursing workloads have to keep additional reserve night shift nurses on call for emergencies. Hospital departments with high death rates should appropriately allocate nursing staff in terms of skill, which has been defined as “the proportion of different nursing grades, and levels of qualification, expertise, and experience” [45]. However, there is very little research studying how night shift nurses cope with patient deaths. It is essential to explore how night shift nurses work and operate in response to patient death, to improve clinical nurses’ experiences coping with patient death.

Experiencing patient death led many nurses to be convinced that they must live well and not die. Clinical nurses believed that death means that everything stops being, and one must live well for their loved ones. Chinese people seem to prefer to believe in their own ability rather than the ability of supernatural beings to protect and save them. Confucian philosophy plays an important role in shaping many Chinese people’s ideological thoughts on the current life and afterlife. Confucius mentioned that “one cannot know death without fully living” and emphasized respecting supernatural beings while staying away from them.” Legalists such as representative Han Fei believed in the legal concept of atheism, which promotes solving the specific problems of human society and politics by rule of law and human interpretation. Tao beliefs advocate for following nature's course and living in harmony with other beings. China is fundamentally a Humanist civilization with pragmatic characteristics which value reality. However, the majority of Chinese people still worship their ancestors, historical heroes, and the gods, to achieve greater courage and noble spirit. Nevertheless, this practice is more of a ritual and tradition rather than religious rite.

Clinical nurses expressed that their reluctance to discuss death arose from their perception that death meant the end of everything, rather than feeling that death was too taboo or mysterious to talk about. Previous literature on experiences coping with patient death reported that new graduate nurses were afraid of death and anxious about death [22]. In our study, participants seemed to display more death avoidance than death fear and anxiety, and this avoidance was unrelated to age, experience, or exposure to death. Nurses have a leading role in communication, cooperation, critical care, and end-of-life care among health-care team members. Being unable to openly discuss death-related topics poses challenges for clinical nurses related to their personal and professional roles when providing end-of-life care or advance care planning, which may result in poor quality care [46]. In China, nursing education and clinical training have focused on disease-related nursing procedures and treatments, and social and cultural attitudes impede palliative care and palliative care education [47]. Public education on hospice care, dying, and death has been scarce [48]. Even among the 15 respondents who were clinical nurses, only one was trained in hospice care and none had education on death management. Previous literature has suggested that there is a lack of planning and strategies for supporting clinical nurses in public hospitals after the death of their patients [25]. To help clinical nurses manage their feelings of sorrow in death or dying-related conversations, in addition to improving emotional resiliency [49, 50], knowledge, and skills in talking about sensitive topics, hospitals need to provide a supportive working environment and sufficient emotional support. Furthermore, they should have dedicated personnel responsible for education and guidance related to coping with patient death.

Clinical nurses often prioritize life-extending treatments. They focus on treating dying patients, recording the signs and symptoms, and responding to changes in the patient’s condition. This is different from a previous study showing that nurses place more emphasis on patients’ wish fulfillment and physical comfort [19]. This may be due to influence by the deep-rooted mission of hospitals to save lives. The characteristics of alleviating suffering and implementing comprehensive and compassionate care are important in determining the quality of end-of-life care and death. In the latest 2015 Quality of Death Index, mainland Chinese residents ranked 71st among 80 countries/regions [51]. This poor ranking should motivate Chinese authorities to improve hospice and palliative care, by raising awareness of “good death” services and fostering a coordinated care environment to meet the care needs of China’s rapidly growing aging population. Within the context of the explosion of sophisticated medical technology and increased hospital admission rates [2, 3], “good death” decisions must be embedded throughout end-of-life care in clinical settings, to aid and enhance a positive death experience for the patient and their loved ones [52].

## Limitations

Some limitations exist of our study exist. All participants worked in the emergency ward, oncology department, and intensive care units; these departments with higher mortality rates were recommended by the leaders of the hospital management department. The semi-structured interview procedure was implemented without pretest interviews, but the interviewer received interview skills training before this study and had experience in qualitative research. Death is a topic based on culture and personal beliefs, so different personal experiences may result in varied opinions. Future research should classify the causes of death faced by nurses to explore further the experiences and challenges of nurses coping with death.

## Conclusions

In the Chinese traditional cultural context, clinical nurses’ negative emotional experiences are shaped by intense feelings of extended filial love and charity. In addition to improving nurses' emotional resiliency, departments with a high mortality rate should optimize the night shift schedule, provide nurses with a reliable and safe working environment, and offer timely technical and psychological support as hospitals encounter increasing admissions and patient death. Chinese authorities should endeavor to foster an awareness of “good death” among clinical health care workers, to embed “good death” decisions grounded in Chinese ethical and moral traditions throughout hospital care for terminally ill patients and their loved ones.

## Supplementary Information

**Additional file 1.** Consolidated criteria for reporting qualitative studies (COREQ):32-item checklist.

## Data Availability

The data that support the findings of this study are available from the first author (Zhang Jinxin, Email: zjx618@mail.sdu.edu.cn), upon reasonable request.
