# Supplementary material for: The experiences of clinical nurses coping with patient death in the context of rising hospital deaths in China: a qualitative study
Source: BMC Palliat Care. 2022 Sep 22;21:163. doi: 10.1186/s12904-022-01054-8 (PMC9494800; doi:10.1186/s12904-022-01054-8)
Supplement: Supplementary file 1 — Additional file 1. Consolidated criteria for reporting qualitative studies (COREQ):32-item checklist. [file 12904_2022_1054_MOESM1_ESM.docx]

**Consolidated criteria for reporting qualitative studies (****COREQ): 32-item checklist**

| **No Item** | **Guide questions/description** | **Reported on Page No.** |
| --- | --- | --- |
| **Domain** **1: Research team and reflexivity** | | |
| **Personal Characteristics** | | |
| 1. Interviewer/facilitator | Which author/s conducted the interview or focus group? | Described at Page 6 |
| 2. Credentials | What were the researcher’s credentials? E.g. PhD, MD | Described at Page 7 |
| 3. Occupation | What was their occupation at the time of the study? | Described at Page 7 |
| 4. Gender | Was the researcher male or female? | Female. |
| 5. Experience and training | What experience or training did the researcher have? | Described at Page 7 |
| **Relationship with participants** | | |
| 6. Relationship established | Was a relationship established prior to study commencement? | Described at Page 5 |
| 7. Participant knowledge of the interviewer | What did the participants know about the researcher? *e.g. personal goals, reasons for doing the research.* | Described at Page 6 |
| 8. Interviewer characteristics | What characteristics were reported about the interviewer/facilitator? *e.g. Bias, assumptions, reasons and interests in the research topic.* | Described at Page 7 |
| **Domain 2: study design** | | |
| **Theoretical framework** | | |
| 9. Methodological orientation and Theory | What methodological orientation was stated to underpin the study? e.g. grounded theory, discourse analysis, ethnography, phenomenology, content analysis. | Described at Page 7 |
| **Participant selection** | | |
| 10. Sampling | How were participants selected? e.g. purposive, convenience, consecutive, snowball | Described at Page 5 |
| 11. Method of approach | How were participants approached? e.g. face-to-face, telephone, mail, email | Described at Page 5 |
| 12. Sample size | How many participants were in the study? | Described at Page 8 |
| 13. Non-participation | How many people refused to participate or dropped out? Reasons? | Not participants refused to participate. |
| **Setting** | | |
| 14. Setting of data collection | Where was the data collected? e.g. home, clinic, workplace | Described at Page 7 |
| 15. Presence of non-participants | Was anyone else present besides the participants and researchers? | Described at Page 6 |
| 16. Description of sample | What are the important characteristics of the sample? e.g. demographic data, date | Table 1. |
| **Data collection** | | |
| 17. Interview guide | Were questions, prompts, guides provided by the authors? Was its pilot tested? | Described at Page 6 |
| 18. Repeat interviews | Were repeat interviews carried out? If yes, how many? | Not. |
| 19. Audio/visual recording | Did the research use audio or visual recording to collect the data? | Yes.  Described at Page 7 |
| 20. Field notes | Were field notes made during and/or after the interview or focus group? | Yes  Described at Page 6 |
| 21. Duration | What was the duration of the interviews or focus group? | Described at Page 8 |
| 22. Data saturation | Was data saturation discussed? | Yes.  Described at Page 5 |
| 23. Transcripts returned | Were transcripts returned to participants for comment and/or correction? | Yes.  Described at Page 7 |
| **Domain 3: analysis and findings** | | |
| **Data analysis** | | |
| 24. Number of data coders | How many data coders coded the data? | Two researchers coded the data.  Described at Page 7 |
| 25. Description of the coding tree | Did authors provide a description of the coding tree? | Table 2. |
| 26. Derivation of themes | Were themes identified in advance or derived from the data? | Described at Page 7 |
| 27. Software | What software, if applicable, was used to manage the data? | Yes.  Microsoft word 16.19 was used to manage the data. |
| 28.Participant checking | Did participants provide feedback on the findings? | Yes.  Page 7 |
| **Reporting** |  |  |
| 29. Quotations presented | Were participant quotations presented to illustrate the themes / findings? Was each quotation identified? e.g. participant number | Yes.  Page 10 to 15 |
| 30. Data and findings consistent | Was there consistency between the data presented and the findings? | Page 10 to 15 and Table 2 |
| 31. Clarity of major themes | Were major themes clearly presented in the findings? | Page 10 to 15 and Table 2 |
| 32. Clarity of minor themes | Is there a description of diverse cases or discussion of minor themes? | Page 10 to 15 and Table 2 |

Developed from: Tong A, Sainsbury P, Craig J. Consolidated criteria for reporting qualitative research (COREQ): a 32-item checklist for interviews and focus groups. International Journal for Quality in Health Care. 2007. Volume 19, Number 6: pp. 349 – 357
